# Inhibition of GRP78 abrogates radioresistance in oropharyngeal carcinoma cells after EGFR inhibition by cetuximab

**DOI:** 10.1371/journal.pone.0188932

**Published:** 2017-12-12

**Authors:** Chaonan Sun, Chuyang Han, Yuanjun Jiang, Ning Han, Miao Zhang, Guang Li, Qiao Qiao

**Affiliations:** 1 Department of Radiotherapy, the First Hospital of China Medical University, Shenyang, Liaoning, China; 2 Department of Urology, the First Hospital of China Medical University, Shenyang, Liaoning, China; Northwestren University, UNITED STATES

## Abstract

The EGFR-specific mAb cetuximab is one of the most effective treatments for oropharyngeal carcinoma, while patient responses to EGFR inhibitors given alone are modest. Combination treatment with radiation can improve the efficacy of treatment through increasing radiosensitivity, while resistance to radiation after administration of cetuximab limits its efficiency. Radiation and drugs can damage the endoplasmic reticulum (ER) homeostatic state and result in ER stress (ERS), subsequently causing resistance to radiation and drugs. Whether the ERS pathway is involved in radioresistance after administration of cetuximab has not been reported. Herein, we show that cetuximab could increase the radiosensitivity of FaDu cells but not Detroit562 cells. In addition, cetuximab inhibited the radiation-induced activation of the ERS signalling pathway IRE1α/ATF6-GRP78 in FaDu cells, while this effect was absent in Detroit562 cells. Silencing GRP78 increased the radiosensitivity of oropharyngeal carcinoma cells and inhibited radiation-induced DNA double-strand-break (DSB) repair and autophagy. More interestingly, silencing GRP78 abrogated resistance to cetuximab and radiation in Detroit562 cells and had a synergistic effect with cetuximab in increasing the radiosensitivity of FaDu cells. Immunohistochemistry showed that overexpression of both GRP78 and EGFR was associated with a poor prognosis in oropharyngeal carcinoma patients (P<0.05). Overall, the results of this study show that radioresistance after EGFR inhibition by cetuximab is mediated by the ERS signalling pathway IRE1α/ATF6-GRP78. This suppression was consequently unable to inhibit radiation-induced DSB repair and autophagy in oropharyngeal carcinoma cells, which conferred resistance to radiotherapy and cetuximab. These results suggest that the cooperative effects of radiotherapy and cetuximab could be further improved by inhibiting GRP78 in non-responsive oropharyngeal carcinoma patients.

## Introduction

The incidence of oropharyngeal carcinoma has increased in recent years [1]. Human papilloma virus (HPV) infection is an important cause of oropharyngeal carcinoma and is also implicated in cancer prognosis. The prognosis of HPV (+) oropharyngeal carcinoma patients was significantly better than that of HPV (-) patients after radical radiotherapy, suggesting that HPV (+) patients have higher intrinsic radiosensitivity than HPV (-) patients [2]. Therefore, it is of great urgency to increase the radiosensitivity of HPV (-) oropharyngeal carcinoma to improve the efficacy of radiotherapy. EGFR is overexpressed in many malignancies, and its overexpression is associated with tumour radioresistance [3, 4]. Therefore, therapies targeting EGFR can increase radiosensitivity and improve the prognosis of cancer after radiotherapy. The EGFR-specific mAb cetuximab combined with radiotherapy has been shown to improve the median survival of patients with head and neck cancer to 49 months, compared with that of 29.3 months in patients treated with radiotherapy alone [5]. However, cetuximab improves the efficacy of radiotherapy in only a subgroup of patients with head and neck squamous cell carcinoma (HNSCC), with 50% of patient still experiencing local recurrence [6], and EGFR levels cannot predict the efficacy of cetuximab combined with radiotherapy [7]. It is therefore essential to explore the mechanism underlying the resistance to radiation after administration of cetuximab for proper patient selection and for improvement of treatment efficacy.

Radiation, drugs and other stimuli can cause DNA damage and induce endoplasmic reticulum (ER) stress (ERS), while sustained ERS protects cells from death and induces treatment resistance via regulation of the expression of apoptosis- and cell cycle-related proteins [8]. Our previous study showed that the ERS signalling pathway protein kinase RNA-like endoplasmic reticulum kinase (PERK) regulated radioresistance in oropharyngeal carcinoma through NF-kB-mediated phosphorylation of eukaryotic initiation factor-2 (eIF2α), enhancing X-ray-induced activation of DNA DSB repair, cell apoptosis inhibition and G2/M cell cycle arrest [9]. GRP78/BiP, a central mediator of ERS, is involved in the regulation of a variety of biological functions, including protein folding, ER calcium binding and control of the activation of transmembrane ER stress sensors [10]. GRP78 is closely related to tumour proliferation and metastasis and is also closely associated with tumour chemotherapy and radiotherapy resistance [11]. Recently, GRP78 expression was found to be elevated in many tumours and cancer cell lines, including head and neck cancer [12], and GRP78 overexpression is associated with poor prognosis in head and neck tumours [13].

It has been reported that EGF can induce cell proliferation through activation of the ERS signalling pathway [14]. We therefore hypothesized that resistance to the combination of cetuximab and radiation may be related to changes in the stress response pathways after irradiation. We first demonstrated, at the cellular level, that cetuximab could inhibit radiation-induced ERS to regulate the radiosensitivity of oropharyngeal carcinoma cells and elucidated the underlying pathways and mechanisms of action. We further silenced the ERS chaperone GRP78 and explored its role in cetuximab-mediated radiosensitization. Finally, we utilized the histological specimens of patients with HPV (-) oropharyngeal carcinoma, analysed the correlation between EGFR and ERS sensor proteins and determined the correlation between EGFR and GRP78 signalling pathway activation and oropharyngeal carcinoma prognosis after radical radiotherapy. This study aimed to explore the targets of cetuximab and radiation resistance and to propose new treatments for patients who are resistant to cetuximab combined with radiotherapy.

## Materials and methods

### Cell culture, transfection and reagents

The human oropharyngeal squamous cell carcinoma cell lines FaDu and Detroit562 were purchased from the ATCC (Manassas, VA, USA) and cultured in MEM containing 10% heat-inactive foetal bovine serum, 100 U/mL penicillin and 100 μg/mL streptomycin.

ON-TARGETplus SMARTpool siRNAs for GRP78, PERK, inositol-requiring enzyme-1 (IRE-1), activating transcription factor-6 (ATF6) and ON-TARGETplus non-targeting siRNA #1 were purchased from Dharmacon (ThermoFisher Scientific, USA). The cells were transfected with small interfering RNA (siRNA) using the DharmaFECT 1 transfection reagent from Dharmacon.

The inhibitor of DSB repair Ly294002 and the autophagy inhibitor 3- Methyladenine (3-MA) were purchased from Sigma (Sigma Chemical, St Louis, MO, USA). The anti-EGFR antibody cetuximab was purchased from Merck (Merck, Darmstadt, Germany).

### Colony survival experiment

Pre-treated cells were inoculated onto 6-well plates at gradient concentrations ranging from 10^2^ to 10^4^ cells. The cells were then irradiated using a SIEMENS linear accelerator (SIEMENS Medical Systems, Germany). The doses were 0, 2, 4 and 6 Gy. The dose rate was 2 Gy/min. The cells were continuously cultured, and the number of colonies in samples with more than 50 cells was counted after 10–14 days. The data were fitted into the classic multitarget single hit model: SF = 1- (1- e^-D/D0^)N to generate the dose-survival curve. And the mean lethal dose (D0), quasi-threshold dose (Dq), survival fraction at 2 Gy (SF2), and sensitivity enhancement ratio (SER) (SER = D0 control group/D0 combination group) were calculated.

### Western blot analysis

Total cellular proteins were extracted using lysis buffer (Pierce, Rockford, IL, USA). The method applied has been described previously [15]. Membranes were incubated with primary antibodies, including those against PDI, Ero1-Lα, ERP57, phospho-eIF2α, phospho-ATM, Bcl-2, DNA-PK, LC3B, Atg16L1 and cleaved poly(ADP-ribose) polymerase (PARP), β-actin (1:1000; Cell Signaling Technology, USA), GRP78, IRE-1, ATF6 and PERK (1:1000; Abcam, USA) at 4°C overnight. Secondary antibodies, including anti-mouse and anti-rabbit IgG antibodies (1:1000 dilution; Cell Signaling Technology, USA) at room temperature for 2 h. Target proteins on PVDF membranes were visualized with LumiGLO (Cell Signaling Technology, USA) and captured using a DNR Bio Imaging System (DNR, Israel).

### RNA preparation and real-time quantitative PCR

RNA extraction was performed according to the protocol supplied with the RNeasy Mini reagent kit (Qiagen, Valencia, CA, USA). Reverse transcription was performed using the M-MLV reverse transcription reagent kit (Invitrogen, Carlsbad, CA, USA). Quantitative analyses of the mRNA expression levels of GRP78 were performed using the TaqMan analysis system (Applied Biosystems, Carlsbad, CA, USA). For GRP78, the forward primer was 5’- GAACACAGTGGTGCCTACCAAGAA -3’ and the reverse primer was 5’- TCCAGTCAGATCAAATGTACCCAGA -3’; for β-actin, the forward primer was 5’-TGGCACCCAGCACAATGAA-3’ and the reverse primer was 5’-CTAAGTCATAGTCCGCCTAGAAGCA -3’.

### Immunofluorescence

Pre-treated cells received 5 Gy radiation. Cells were collected after 1 h of 5Gy radiation, evenly smeared onto slides. Slides were then incubated with primary anti-γ-H2AX and LC3B (1:500; Cell Singling Technology, USA) antibody at 4°C overnight. The method applied has been described in detail in our previous study [9].

### CCK-8 assay

Cell proliferation was analysed using a Cell Counting Kit-8 (CCK-8) kit (Dojindo, Gaithersburg, MD, USA) according to the manufacturer’s protocol. The method applied has been described in detail in our previous study [16].

### Flow cytometry

Experiments were performed according to the protocol supplied with the Annexin-Green Apoptosis cell detection reagent kit (Cell Signaling Technology, USA). The percentage of apoptotic cells was detected using a FACScan flow cytometer (FACSCalibur BD; BD Biosciences, San Jose, CA, USA). The specific experimental methods have been described previously [17].

### Immunohistochemistry

Pathological tumour sections were obtained from 80 patients with HPV (-) oropharyngeal squamous cell carcinoma who received radical radiotherapy with or without concurrent chemotherapy in our hospital between 2005 and 2011. All recruited patients provided informed consent.

The method applied has been described in detail in our previous study [16]. The GRP78 primary antibody was purchased from Abcam (1:300 dilution; Abcam, USA) and the EGFR primary antibody was purchased from Santa Cruz (1:50 dilution; Santa Cruz, USA). GRP78 staining was cytoplasmic, and a semi-quantitative scoring criterion was used for GRP78 immunohistochemistry [18]. Tumour samples with a final score ≤ 2 were considered to have negative staining, while tumour samples with a final score ≥ 3 were considered to have positive staining. According to the rating criteria for EGFR [4], EGFR scoring refers to both cytoplasmic and membranous staining.

### Statistical analyses

The data from three independent experiments are expressed as the mean ± standard deviation. The Kaplan-Meier method was used for survival analyses. Comparisons between two groups were performed using the *t* test. *P* value < 0.05 indicated statistical significance. SPSS 13.0 software was used to perform the statistical analyses.

## Results

### Radioresistance after EGFR inhibition by cetuximab is associated with deinhibition of the ERS signalling pathway IRE1α/ATF6-GRP78

**Fig 1:**
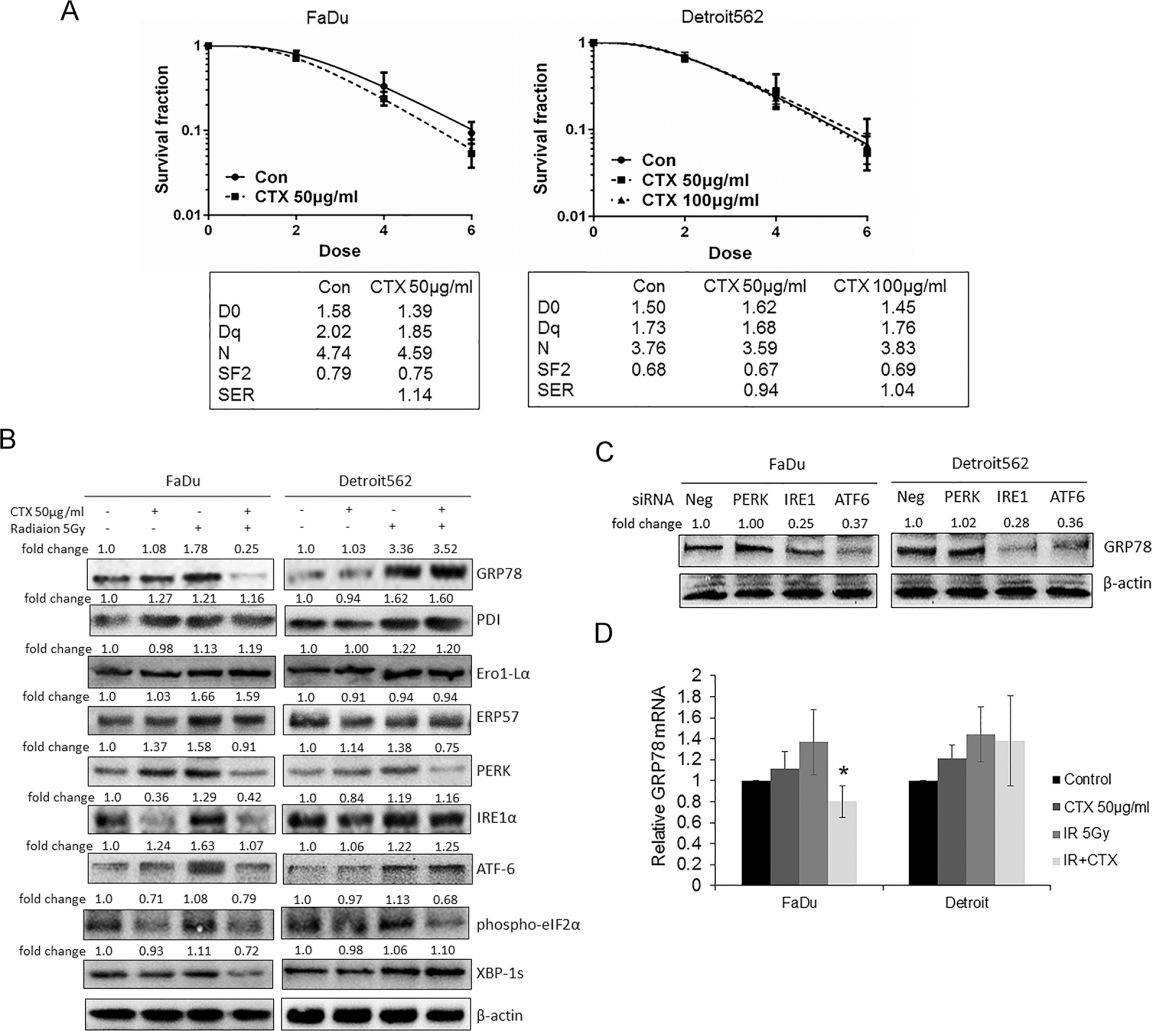
Radiation resistance after treatment with cetuximab is associated with deinhibition of the ERS signalling pathway IRE1α/ATF6-GRP78. (A) Colony formation experiments showed that cetuximab (50 μg/mL, 12 h) decreased the incidence of colony formation in FaDu oropharyngeal carcinoma cells after irradiation but had no significant effect on Detroit562 cells, even when the cetuximab concentration was increased to 100 μg/mL. Radiation parameters were fitted to a classic multitarget single hit model, as shown in the table. (B) Oropharyngeal carcinoma cells were pretreated with cetuximab and then treated with 5 Gy radiation after 12 h. Cells were collected 12 h later for Western blot detection. The results showed that cetuximab inhibited the radiation-induced expression of GRP78, IRE1α, ATF6 and XBP-1s proteins in FaDu cells but had no such effects on Detroit562 cells. Cetuximab inhibited the expression of PERK and phospho-eIF2α protein in FaDu and Detroit562 cells. Radiotherapy partially induced increases in the expression of PDI, Ero1-Lα and ERP57, while cetuximab exerted no significant effect on PDI, Ero1-Lα or ERP57 expression. Bands were quantified using ImageJ software and were normalized to a loading control. Fold changes are shown compared with the negative control lane without radiation. N/A = not applicable. (C) siRNAs were transfected into cells to silence PERK, IRE1α or ATF6. Western blot results showed that silencing IRE1α and ATF6 inhibited the expression of GRP78 protein in oropharyngeal carcinoma cells, while silencing PERK had no effect on GRP78 protein expression. (D) After treatment with 50 μg/mL cetuximab for 12 h, oropharyngeal carcinoma cells received 5 Gy irradiation, and the cells were harvested after 1 h. RT-PCR results showed that cetuximab inhibited the radiation-induced expression of GRP78 mRNA in FaDu oropharyngeal carcinoma cells but had no significant effects on Detroit562 cells. Compared with the IR group, *P < 0.05. Note: CTX = Cetuximab, Con = Control, Neg = Negative.

Previous studies have revealed that cetuximab can increase the radiosensitivity of head and neck tumours [7]. We pretreated FaDu and Detroit562 oropharyngeal carcinoma cells with 50 μg/mL cetuximab, and the results of the colony formation assay showed that cetuximab increased the radiosensitivity of FaDu cells, with a radiation sensitization ratio of 1.14, but showed no radiosensitization effects in Detroit562 cells. Based on the finding by González JE et al. that the radiosensitization effect of cetuximab was dependent on drug concentrations [19], we further increased the cetuximab concentration to 100 μg/mL. Again, the result showed that cetuximab did not present radiosensitization effects in Detroit562 cells (Fig 1A), indicating that Detroit562 cells are intrinsically resistant to radiation after cetuximab.

Radiation can induce ERS and upregulate ER chaperones to facilitate cell survival. Therefore, we hypothesized that the cetuximab-mediated radiosensitization effects in oropharyngeal carcinoma cells may be associated with changes in ERS after radiation. Interestingly, cetuximab inhibited the radiation-induced expression of GRP78 protein in FaDu cells but did not show this effect in Detroit562 cells. We further detected the expression of the ERS chaperones protein disulfide isomerase (PDI), Ero1-Lα and ERP57, and the results showed that radiation partially induced increases in PDI, Ero1-Lα and ERP57 expression, whereas further administration of cetuximab showed no significant effects on PDI, Ero1-Lα or ERP57 expression.

ERS constitutes the UPR via activation of PERK-eIF2α, IRE1- XBP-1s and ATF6 signalling pathways. Therefore, we examined the expression of PERK, eIF2α, IRE1α, XBP-1s and ATF6 proteins to determine which pathway was involved in the radiosensitization effect of cetuximab. Similar to the effects of cetuximab on GRP78 protein expression, cetuximab inhibited radiation-induced expression of IRE1α and ATF6 proteins in FaDu cells but not in Detroit562 cells. In contrast, cetuximab inhibited radiation-induced PERK and eIF2α protein expression in both cell lines (Fig 1B). These results suggested that cetuximab regulates radiation-induced expression of IRE1α, XBP-1s and ATF6, which in turn inhibit GRP78 protein expression. To confirm this inference, we transfected cells with siRNAs that silenced the protein expression of PERK, IRE1α and ATF6. Silencing IRE1α and ATF6 inhibited GRP78 protein expression, while silencing PERK had no significant effect on GRP78 protein expression (Fig 1C). In addition, RT-PCR experiments showed that cetuximab inhibited radiation-induced expression of GRP78 mRNA in FaDu cells (P < 0.05) but did not have significant effects in Detroit562 cells (Fig 1D). Together, we demonstrated that resistance to radiation after administration of cetuximab is associated with the deinhibition of IRE1α/ATF6-GRP78.

### Radiosensitization of cetuximab is associated with inhibition of persistent overexpression of the radiation-induced ERS chaperone GRP78

**Fig 2:**
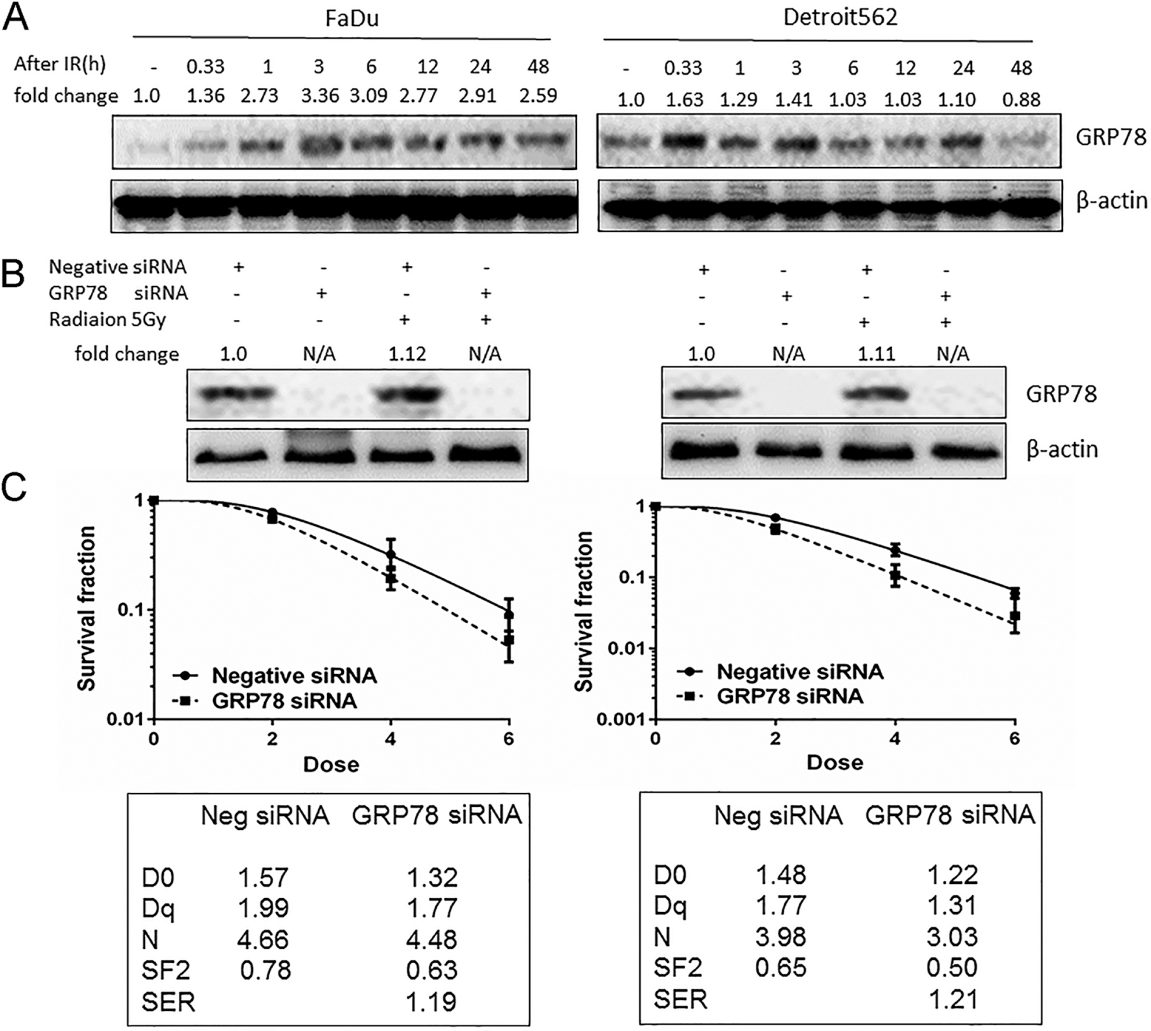
Cetuximab enhances radiosensitivity by inhibiting the radiation-induced ERS chaperone GRP78. (A) Western blot results showed that irradiation (5 Gy) induced the activation of GRP78 in oropharyngeal carcinoma cells in a time-dependent manner. (B) Western blot results showed that silencing GRP78 inhibited the expression of GRP78 protein in oropharyngeal carcinoma cells. (C) Silencing GRP78 reduced the colony formation rate of oropharyngeal carcinoma cells. Additionally, radiation parameters were fitted to a classic multitarget single hit model as shown in the table. For (A) and (B), bands were quantified using ImageJ software and were normalized to a loading control. Fold changes are shown compared with the control lane. N/A = not applicable.

The results of present study showed that radiation induced increased expression of GRP78 in oropharyngeal carcinoma cells in a time-dependent manner (Fig 2A). The expression of GRP78 protein was increased 20 min after irradiation, and its expression in FaDu cells peaked at 3 h and was sustained at high levels until 48 h after irradiation. In contrast, the expression of GRP78 protein in Detroit562 cells peaked at 20 min and decreased at 48 h after irradiation. We hypothesized that the radiosensitizing effect on FaDu cells by cetuximab may be due to the sustained effective inhibition of radiation-induced GRP78 overexpression. In contrast, in Detroit562 cells, cetuximab had no obvious effect on the radiation-induced activation of GRP78 protein, which decreased within a short time frame. In addition, the intrinsic radiosensitivity of FaDu cells (D_0_ = 1.58) was lower than that of Detroit562 cells (D_0_ = 1.50). Therefore, the activation of GRP78 protein and the sustained overexpression of GRP78 protein may be involved in the radiation resistance of oropharyngeal carcinoma cells.

To confirm this hypothesis, we transfected cells with siRNA to silence GRP78 (Fig 2B). The results showed that silencing GRP78 increased the radiosensitivity of FaDu and Detroit562 cells, and the radiosensitization ratios (SER) of FaDu and Detroit562 cells were 1.19 and 1.21, respectively (Fig 2C), suggesting that the radiosensitization effect of cetuximab was related to its inhibition of radiation-induced sustained overexpression of GRP78.

### The radioresistant effect of GRP78 is associated with increased radiation-induced DNA double-strand break repair and autophagy as well as subsequent inhibition of apoptosis

**Fig 3:**
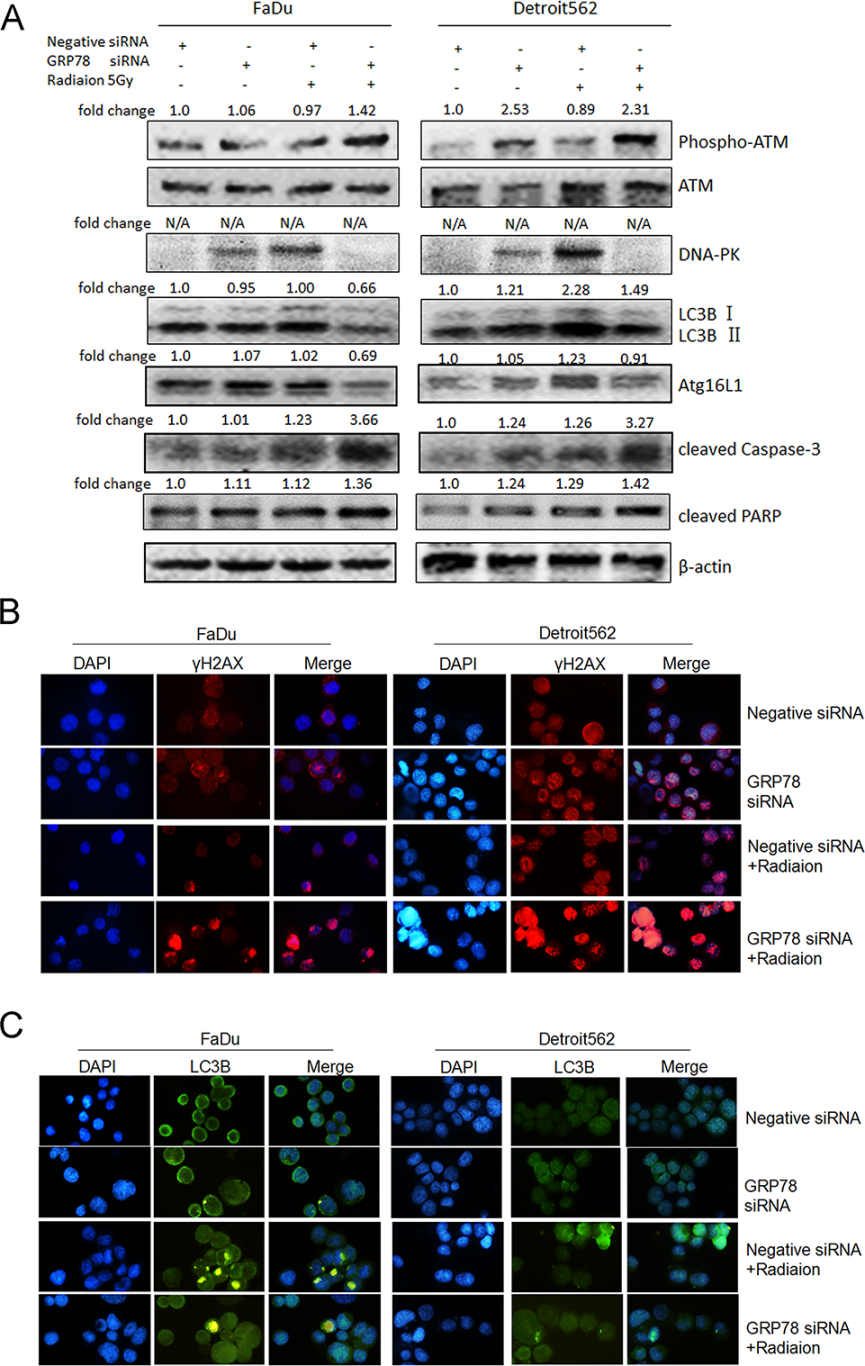
GRP78 confers radioresistance by increasing radiation-induced DNA double-strand break repair and cell autophagy and the subsequent inhibition of apoptosis. (A) Silencing GRP78 inhibited the radiation-induced (5 Gy, 12 h) expression of the DNA double-strand break repair protein DNA-PK and increased the phosphorylation level of ATM. Silencing GRP78 also inhibited the radiation-induced expression of the autophagy-related proteins LC3B (LC3B-II/β-actin) and Atg16L1 and increased the expression of the apoptosis marker protein cleaved caspase-3 and cleaved PARP. (B) Immunofluorescence studies showed that after oropharyngeal carcinoma cells received 5 Gy radiation for 1 h, the γ-H2AX foci in nucleus increased (the blue background indicates the cell nucleus, and light red dots indicate γ-H2AX foci). In addition, the effect of radiation after GRP78 silencing was more evident than that of simple radiation. (C) Immunofluorescence studies by LC3B staining also showed autophagy regions in the nucleus after 5 Gy radiation for 1 h (the blue background indicates the cell nucleus, and light green dots indicate LC3B foci), which was reversed after GRP78 silencing. The oropharyngeal carcinoma cells were pretreated with 20 μmol/L Ly294002 or 5 mmol/L 3-MA for 12 h. The cells were then treated with 5 Gy of radiation. Compared with the IR group, *P < 0.05. For (A), bands were quantified using ImageJ software and were normalized to a loading control. Fold changes are shown compared with the negative control lane without radiation. N/A = not applicable.

It was reported that an EGFR-targeted inhibitor could inhibit DNA double-strand break repair and autophagy to increase radiosensitivity in malignancies [20]. Therefore, we hypothesized that cetuximab may inhibit radiation-induced GRP78 expression and thereby regulate DNA DSBs and autophagy. Western blot analysis showed that silencing GRP78 inhibited the radiation-induced expression of the DNA double-strand break repair protein DNA-PK and increased the phosphorylation level of ATM. In addition, silencing GRP78 also inhibited the protein expression of the autophagy marker LC3B and the related protein Atg16L1 (Fig 3A). In addition, immunofluorescence studies clearly showed DNA DSB regions in the nucleus were characterized by the formation of γ-H2AX foci, which are markers of DSB damage in DNA after radiation. Further, the effect of radiation after GRP78 silencing was more evident than that of simple radiation (Fig 3B). Immunofluorescence studies by LC3B staining also showed autophagy regions in the nucleus after radiation, which was reversed after GRP78 silencing (Fig 3C).

**Fig 4:**
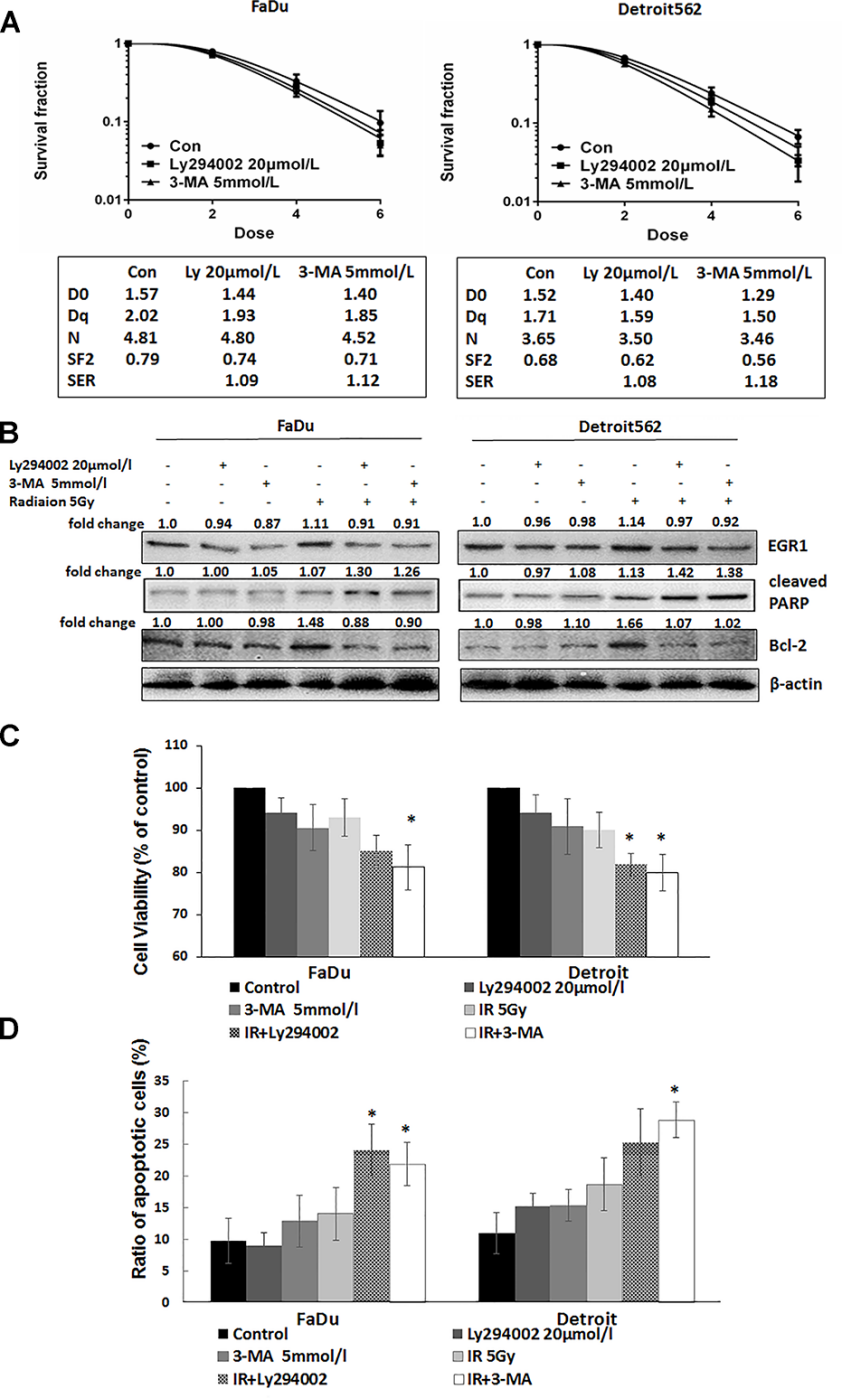
The inhibition of DNA DSB repair and autophagy can increase radiosensitivity in oropharyngeal carcinoma cells by inhibiting cell proliferation and inducing apoptosis. Oropharyngeal carcinoma cells were pretreated with 20 μmol/L Ly294002 or 5 mmol/L 3-MA for 12 h. The cells were then treated with different doses of radiation. (A) The results of cloning showed that Ly294002 and 3-MA could increase the radiosensitivity of oropharyngeal carcinoma cells. Additionally, radiation parameters were fitted to a classic multitarget single hit model as shown in the table. (B) Western blot analysis showed that Ly294002 and 3-MA increased the radiation-induced protein expression of cleaved PARP and inhibited the radiation-induced expression of the anti-apoptotic protein Bcl-2 and the cell proliferation regulatory protein EGR1. (C) At 48 h after irradiation, CCK-8 analysis showed that Ly294002 and 3-MA further reduced the proliferation of cells inhibited by radiation alone. (D) At 48 h after irradiation, apoptosis was detected by flow cytometry after Annexin V/PI staining. The results showed that Ly294002 and 3-MA increased radiation-induced apoptosis. For (C) and (D), compared with the IR group, *P < 0.05. For (B), bands were quantified using ImageJ software and were normalized to a loading control. Fold changes are shown compared with the negative control lane without radiation. N/A = not applicable.

In addition, silencing GRP78 increased the radiation-induced expression of the apoptosis marker protein cleaved caspase-3 and cleaved PARP (Fig 3A). Our previous studies showed that apoptosis is an important mechanism in the regulation of radiosensitivity [15, 17] and that inhibition of DNA double-strand break repair can increase radiation-induced apoptosis [21]. Furthermore, inhibition of autophagy increases radiation-induced tumour cell apoptosis [22]. Therefore, we hypothesize that the increased radiosensitivity of oropharyngeal carcinoma cells upon silencing GRP78 is related to radiation-induced autophagy and DNA double-strand break repair. To confirm this hypothesis, we used the broad-spectrum inhibitor of DNA double-strand break repair, Ly294002, and the autophagy inhibitor, 3-MA, to treat oropharyngeal carcinoma cells. The results of cloning showed that Ly294002 and 3-MA could increase the radiosensitivity of oropharyngeal carcinoma cells. The results showed that Ly294002 and 3-MA inhibited the radiation-induced expression of the cell proliferation regulatory protein EGR1 and anti-apoptotic protein Bcl-2, while they increased the radiation-induced expression of the apoptosis marker protein cleaved PARP (Fig 4B). The results of the CCK-8 analysis also showed that Ly294002 and 3-MA further reduced the proliferation of cells inhibited by radiation alone (Fig 4C). Flow cytometry also demonstrated that Ly294002 and 3-MA increased radiation-induced apoptosis (Fig 4D). These results confirmed that the inhibition of radiation-induced DNA double-strand break repair and autophagy and a subsequent increase in apoptosis might mediate the radiosensitization effect of GRP78 silencing in oropharyngeal carcinoma cells.

### Targeting GRP78 abrogates resistance to radiation after EGFR inhibition by cetuximab

**Fig 5:**
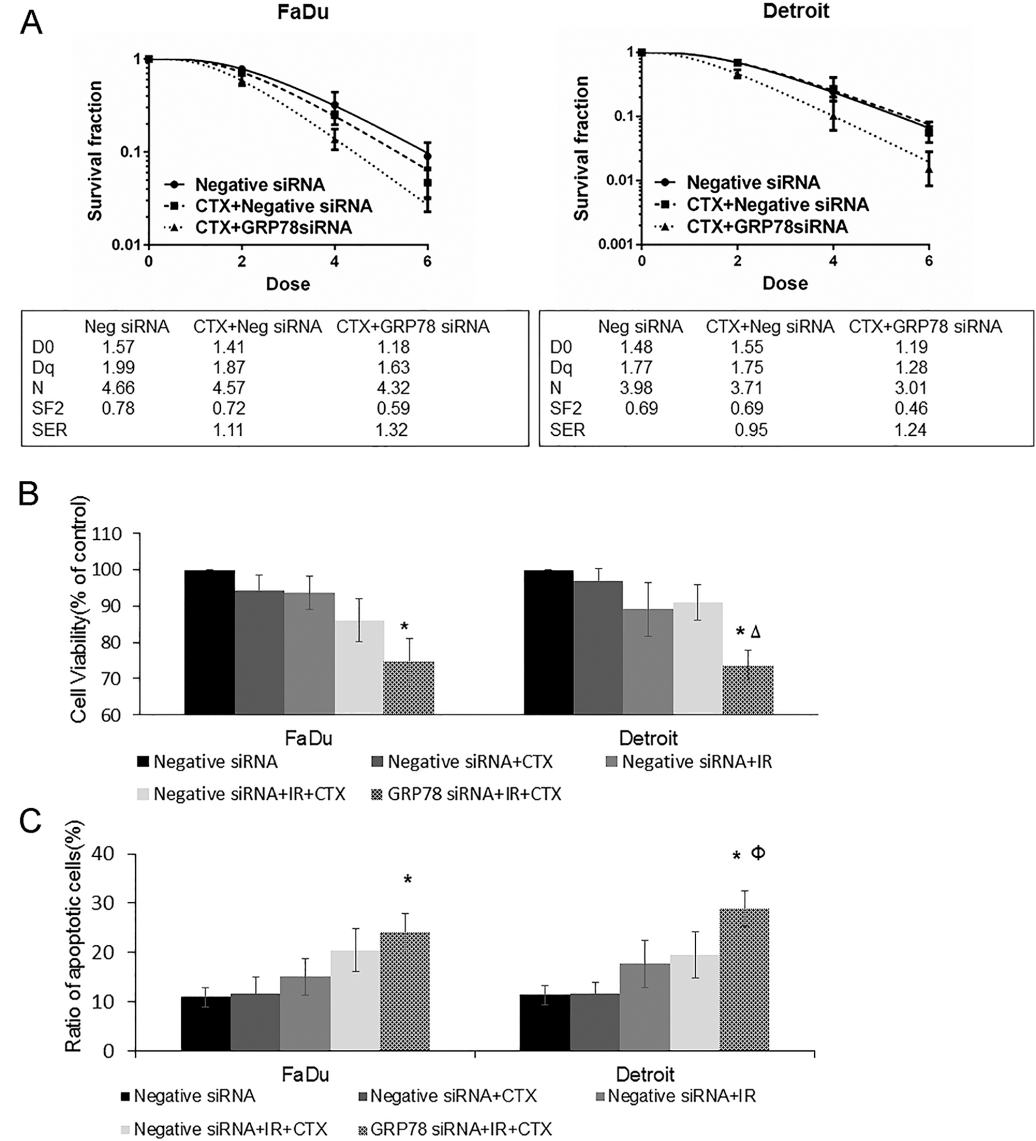
Targeting GRP78 abrogates resistance to cetuximab and radiation. Oropharyngeal carcinoma cells transfected with siRNA to silence GRP78 or negative siRNA were treated with 50 μg/mL cetuximab for 12 h and then irradiated. (A) Colony formation experiments showed that cetuximab inhibited the colony formation of FaDu cells, which was further enhanced by silencing GRP78. In contrast, cetuximab alone did not affect the colony formation of Detroit562 cells, which was reversed by the silencing of GRP78. (B) Cetuximab weakened the radiation-mediated inhibition of FaDu cell proliferation, which was further enhanced by the silencing of GRP78. In addition, cetuximab had no significant effects on the radiation-mediated inhibition of Detroit562 cell proliferation, which was reversed by the silencing of GRP78. (C) Cetuximab increased the radiation-induced apoptosis of FaDu cells, which was further enhanced by the silencing of GRP78. The effect of cetuximab on the apoptosis of Detroit562 cells was not obvious, and this changed after GRP78 was silenced. Note: CTX = cetuximab, Neg = negative. *P < 0.05 Compared with Negative siRNA + IR; ΔP < 0.01 Compared with Negative siRNA + IR + CTX; ФP = 0.05 Compared with Negative siRNA + IR + CTX.

Previous studies had shown that intrinsic resistance to radiation after administration of cetuximab might be due to deinhibition of radiation-induced expression of GRP78; therefore, we speculated that GRP78 may serve as a target. The results of the colony survival assay showed that silencing GRP78 abrogated the radioresistance in Detroit562 cells after treatment with cetuximab, and the SER was 1.24. Interestingly, silencing GRP78 further increased the radiosensitization effect of cetuximab on FaDu cells; the SERs were 1.11 and 1.32, respectively (Fig 5A).

The results of the CCK-8 assay showed that compared with radiation alone, radiation combined with cetuximab led to inhibition of FaDu cell proliferation but had no significant effects on Detroit562 cells. Silencing GRP78 combined with cetuximab treatment significantly inhibited the proliferation of Detroit562 cells, and the effect was more pronounced than radiation alone or radiation combined with cetuximab (P < 0.05 compared with IR; P < 0.01 compared with IR + cetuximab). Interestingly, cetuximab administration after GRP78 silencing led to a more pronounced inhibition of radiation-inhibited FaDu cell proliferation than cetuximab alone (Fig 5B).

We further detected apoptosis and found that cetuximab alone had no effect on radiation-induced apoptosis in Detroit562 cells, whereas silencing of GRP78 led to a significantly increased apoptosis rate compared with radiation alone or radiation combined with cetuximab (P < 0.05 compared with IR; P = 0.05 compared with IR + cetuximab). Cetuximab alone increased radiation-induced FaDu cell apoptosis, and this effect was further enhanced by GRP78 silencing (Fig 5C).

These results suggested that silencing GRP78 could abrogate radioresistance after treatment with cetuximab. Additionally, these findings also suggested that dual inhibition of EGFR (cetuximab) and ERS (GRP78 siRNA) led to greater radiosensitization effects than either cetuximab or GRP78 siRNA alone and indicated that this combination treatment may be clinically relevant even for non-responsive patients with oropharyngeal carcinoma.

### EGFR and the ERS chaperone GRP78 expression in human HPV (-) oropharyngeal squamous cell carcinoma

**Fig 6:**
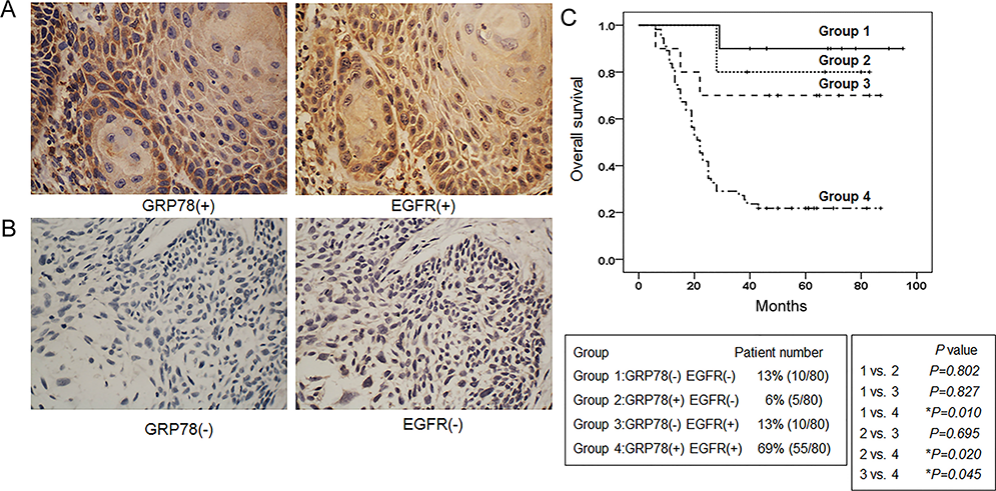
Expression of EGFR and GRP78 in human oropharyngeal carcinoma tissues. Schematic diagrams of (A) high and (B) low EGFR and GRP78 expression in oropharyngeal squamous cell carcinoma tissues. Magnification × 400. (B) Analysis of the correlation between the OS of patients with oropharyngeal carcinoma and the expression of EGFR and GRP78 using the Kaplan-Meier method.

EGFR overexpression is an independent prognostic factor for malignancies [23]. GRP78 overexpression was also associated with a poor prognosis in head and neck carcinoma [12], but the correlation of EGFR and ERS signalling pathways in head and neck squamous cell carcinoma has not been reported in histological studies. Considering the cell lines used in this study were HPV (-) oropharyngeal carcinoma cells, we selected HPV (-) human oropharyngeal carcinoma for the detection of EGFR and GRP78 expression by immunohistochemistry. The results from the Spearman correlation analysis showed that EGFR expression was significantly correlated with GRP78 expression (r = 0.289, p = 0.009) (Fig 6A and 6B). To further clarify the relationship between EGFR and GRP78 expression and the prognosis of patients with oropharyngeal carcinoma, we divided the patients into four groups: EGFR (-) GRP78 (-), EGFR (+) GRP78 (-), EGFR (-) GRP78 (+) and EGFR (+) GRP78 (+). A Kaplan-Meier survival analysis showed that oropharyngeal carcinoma patients with EGFR and GRP78 co-overexpression had the worst prognosis with respect to overall survival (P < 0.05, Fig 6C). The remaining groups contained small numbers of cases, and no significant differences were observed among those groups.

## Discussion

EGFR is overexpressed in head and neck tumours and is associated with radioresistance and poor prognosis [3]. Cetuximab is an EGFR-targeted monoclonal antibody that binds to the EGFR ligand binding domain [24]. *In vitro* and *in vivo* studies have shown that cetuximab increases the radiosensitivity of tumours [25, 26]. However, further studies have shown that compared with concurrent radiochemotherapy, cetuximab combined with radiotherapy does not lead to a significant difference in PFS [27], and 50% of patients still experience local recurrence [6]. Therefore, some patients are still resistant to radiotherapy after treatment with cetuximab. The results of this study showed that cetuximab has radiosensitization effects on FaDu cells but not Detroit562 cells, even if the concentration is increased to 100 μg/mL. Previous studies have suggested several molecular mechanisms, such as the association of K-RAS mutations and acquired RAS or EGFR mutations implicated in cetuximab resistance [28, 29]. At present, the specific mechanism underlying the resistance to radiotherapy after cetuximab in head and neck carcinoma remains unclear.

The ER is the primary site for the regulation of protein synthesis, protein folding and intracellular calcium (Ca^2+^) levels. Stimuli, such as viruses, drugs and radiation, cause the aggregation of misfolded proteins in the ER, leading to ER functional abnormalities and ERS. The induction of GRP78 is widely used as a marker for ERS. GRP78 is overexpressed in malignant tumours [12, 13] and plays an important role in tumour formation, metastasis and invasion [30]. GRP78 is overexpressed in radiation-resistant head and neck tumour cells and is closely related to radioresistance in malignancies [11, 31]. Our results showed that cetuximab inhibited the radiation-induced GRP78 protein expression in FaDu cells, while no effects were observed on the intrinsic resistance of Detroit562 cells to cetuximab and radiation. Our results suggest that cetuximab-mediated radiosensitization is related to the regulation of GRP78 protein expression. Cetuximab had no regulatory effect on radiation-induced expression of two other ERS chaperones, PDI and ERP57. Of the ER chaperones, PDI-like proteins, such as PDI, Erp72 and Erp57, are characterized by the presence of a thioredoxin domain and contain an active-site double-cysteine motif and thus have oxido-reductase activity [32]. GRP78 has no such motif, and the difference in structure may lead to different effects of cetuximab on the radiation-induced expression of the PDI family of proteins (PDI and ERP57) and GRP78. Our study, for the first time, found that the expression of EGFR and GRP78 in oropharyngeal carcinoma tissue was correlated and that the co-expression of these two proteins indicated a poor prognosis.

Under normal circumstances, cells attempt to resolve ERS by activating the UPR [33]. In mammalian cells, the function of the UPR is mediated by three ER-localized transmembrane proteins acting as sensors: PERK, IRE1 and ATF6 [33]. PERK induces p-eIF2α, which in turn suppresses global mRNA translation and favours apoptosis by increasing the translation of ATF4. The cleavage of ATF6 leads to the transcription of genes required to restore ER homeostasis. In addition, activation of IRE1-α leads to the splicing of XBP1, which in turn translocates to the nucleus to activate the transcription of genes involved in the UPR and in cell adaptation [34]. Few studies have been conducted on the regulation of the UPR by cetuximab, and we only found two related reports [35, 36], while no reports have focused on the combination therapy of cetuximab and radiotherapy on the regulation of UPR. Chiara Pozzi et al. [35] showed that cetuximab in combination with chemotherapy triggers immunogenic cell death by blocking IRE1α. Lei et al. [36] showed that cetuximab activates the UPR-associated protein p-eIF2α via regulation of the NLRX1-TUFM protein complex. The results of this study demonstrate that cetuximab increases the radiosensitivity of oropharyngeal carcinoma cells via the inhibition of radiation-induced IRE1α/ATF6-GRP78 and that the silencing of GRP78 abrogates the resistance of Detroit562 cells to cetuximab and radiation. In addition, this study found for the first time that cetuximab and GRP78 have synergistic radiosensitizing effects.

Radiotherapy can induce autophagy, which is the main route of DNA damage repair, and inhibition of autophagy can increase the radiosensitivity of cells. EGFR-targeted therapy increases the radiosensitivity of non-small cell lung cancer through the inhibition of autophagy [20]. Moreover, one mechanism by which the cell survives the UPR is the induction of autophagy [37]. However, controversies remain in the field of autophagy. Some studies suggest that the induction of autophagy can increase the sensitivity of cells to radiotherapy [38], but the specific mechanism is unclear and may be related to tumour type and mode of action. Our study indicates that silencing GRP78 inhibits radiation-induced autophagy; this subsequently leads to increased radiation-induced apoptosis, which regulates the radiosensitivity of oropharyngeal carcinoma cells.

DSBs, which are common incidents during radiotherapy, are repaired by nonhomologous end-joining (NHEJ) and homologous recombination (HR). DNA-PK is a key component of the NHEJ repair pathway, and its small molecule inhibitors increase radiosensitivity by binding to the DNA-PKcs kinase region. The PI3K family member ATM is the core protein that is regulated by the HR pathway. ATM recognizes phosphorylated downstream molecules related to damage and facilitates 10% of post-damage DNA repair [39]. Nickson CM et al. indicated that misregulation of DNA damage repair pathways in HPV-positive head and neck squamous cell carcinoma contribute to cellular radiosensitivity [40]. Mukherjee et al. demonstrated that EGFRvIII induces expression of the DNA double-strand break repair enzyme DNA-PKcs and regulates glioblastoma radiation resistance [41]. The results of this study demonstrated that cetuximab increases oropharyngeal cancer cell radiosensitivity via the inhibition of GRP78 and the subsequent inhibition of DNA NHEJ and HR repair.

In conclusion, this study confirmed for the first time that cetuximab increases the radiosensitivity of oropharyngeal squamous cell carcinoma through inhibition of radiation-induced ERS. We first confirmed at the cellular level that cetuximab increased the radiosensitivity of FaDu cells, while Detroit562 cells exhibited intrinsic resistance to cetuximab and radiation. Cetuximab inhibited radiation-induced activation of the ERS signalling pathway proteins IRE1α/ATF6-GRP78 in FaDu cells but had no such effects in Detroit562 cells, suggesting that radioresistance after EGFR inhibition by cetuximab is associated with IRE1α/ATF6-GRP78 deinhibition. To confirm this hypothesis, we transfected cells with siRNA to silence GRP78, and a subsequent colony formation assay showed that inhibition of GRP78 increased the radiosensitivity of oropharyngeal carcinoma cells. The mechanism of action was that the silencing of GRP78 inhibited radiation-induced cell autophagy and DNA double-strand break repair, which increased radiation-induced apoptosis. In addition, the silencing of GRP78 abrogated radioresistance after treatment with cetuximab. Interestingly, we found that silencing GRP78 further increased the radiosensitization effect of cetuximab in FaDu cells. Finally, it was revealed by immunohistochemistry that the expression levels of EGFR and GRP78 were correlated and that the co-overexpression of EGFR and GRP78 was correlated with a poor prognosis of oropharyngeal carcinoma. These results suggested that the cooperative effects of radiotherapy and EGFR-targeted inhibitor therapy could be further improved by inhibition of GRP78 in non-responsive oropharyngeal carcinoma patients.

## Supporting information

S1 Fig Radiation resistance after treatment with cetuximab is associated with deinhibition of the ERS signalling pathway IRE1α/ATF6-GRP78. (ZIP)

S2 Fig Cetuximab enhances radiosensitivity by inhibiting the radiation-induced ERS chaperone GRP78. (ZIP)

S3 Fig GRP78 confers radioresistance by increasing radiation-induced DNA double-strand break repair and cell autophagy and the subsequent inhibition of apoptosis. (7Z)

S4 Fig The inhibition of DNA DSB repair and autophagy can increase radiosensitivity in oropharyngeal carcinoma cells by inhibiting cell proliferation and inducing apoptosis. (ZIP)

S5 Fig Targeting GRP78 abrogates resistance to cetuximab and radiation. (ZIP)

S6 Fig Expression of EGFR and GRP78 in human oropharyngeal carcinoma tissues. (ZIP)
