# Supplementary figures and images for: Inhibition of GRP78 abrogates radioresistance in oropharyngeal carcinoma cells after EGFR inhibition by cetuximab
Source: PLoS One. 2017 Dec 12;12(12):e0188932. doi: 10.1371/journal.pone.0188932 (PMC5726659; doi:10.1371/journal.pone.0188932)

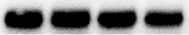

Supplement: S1 Fig — (ZIP) [file pone.0188932.s001.zip › support file fig.1/B/De actin.tif]

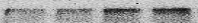

Supplement: S1 Fig — (ZIP) [file pone.0188932.s001.zip › support file fig.1/B/De ATF6.tif]

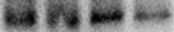

Supplement: S1 Fig — (ZIP) [file pone.0188932.s001.zip › support file fig.1/B/De eIF.tif]

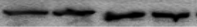

Supplement: S1 Fig — (ZIP) [file pone.0188932.s001.zip › support file fig.1/B/De erol-1a.tif]

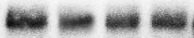

Supplement: S1 Fig — (ZIP) [file pone.0188932.s001.zip › support file fig.1/B/De ERP57.tif]

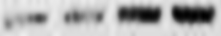

Supplement: S1 Fig — (ZIP) [file pone.0188932.s001.zip › support file fig.1/B/De GRP78.tif]

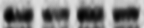

Supplement: S1 Fig — (ZIP) [file pone.0188932.s001.zip › support file fig.1/B/De IRE.tif]

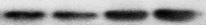

Supplement: S1 Fig — (ZIP) [file pone.0188932.s001.zip › support file fig.1/B/De PDI.tif]

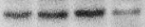

Supplement: S1 Fig — (ZIP) [file pone.0188932.s001.zip › support file fig.1/B/De PERK.tif]

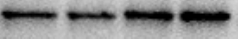

Supplement: S1 Fig — (ZIP) [file pone.0188932.s001.zip › support file fig.1/B/DexBP1s.tif]

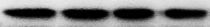

Supplement: S1 Fig — (ZIP) [file pone.0188932.s001.zip › support file fig.1/B/FaDu actin.tif]

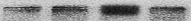

Supplement: S1 Fig — (ZIP) [file pone.0188932.s001.zip › support file fig.1/B/FaDu ATF6.tif]

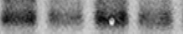

Supplement: S1 Fig — (ZIP) [file pone.0188932.s001.zip › support file fig.1/B/FaDu eIF.tif]

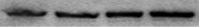

Supplement: S1 Fig — (ZIP) [file pone.0188932.s001.zip › support file fig.1/B/FaDu erol-1a.tif]

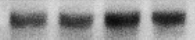

Supplement: S1 Fig — (ZIP) [file pone.0188932.s001.zip › support file fig.1/B/FaDu ERP57.tif]

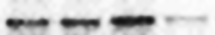

Supplement: S1 Fig — (ZIP) [file pone.0188932.s001.zip › support file fig.1/B/FaDu GRP78.tif]

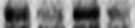

Supplement: S1 Fig — (ZIP) [file pone.0188932.s001.zip › support file fig.1/B/FaDu IRE.tif]

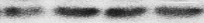

Supplement: S1 Fig — (ZIP) [file pone.0188932.s001.zip › support file fig.1/B/FaDu PDI.tif]

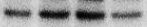

Supplement: S1 Fig — (ZIP) [file pone.0188932.s001.zip › support file fig.1/B/FaDu PERK.tif]

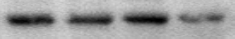

Supplement: S1 Fig — (ZIP) [file pone.0188932.s001.zip › support file fig.1/B/FaDu xBP1s.tif]

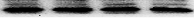

Supplement: S1 Fig — (ZIP) [file pone.0188932.s001.zip › support file fig.1/C/De actin.tif]

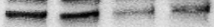

Supplement: S1 Fig — (ZIP) [file pone.0188932.s001.zip › support file fig.1/C/De GRP78.tif]

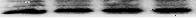

Supplement: S1 Fig — (ZIP) [file pone.0188932.s001.zip › support file fig.1/C/FaDu actin.tif]

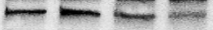

Supplement: S1 Fig — (ZIP) [file pone.0188932.s001.zip › support file fig.1/C/FaDu GRP78.tif]

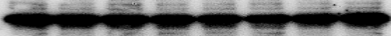

Supplement: S2 Fig — (ZIP) [file pone.0188932.s002.zip › support file fig.2/A/De actin.tif]

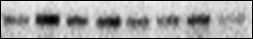

Supplement: S2 Fig — (ZIP) [file pone.0188932.s002.zip › support file fig.2/A/De GRP78.tif]

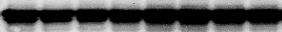

Supplement: S2 Fig — (ZIP) [file pone.0188932.s002.zip › support file fig.2/A/FaDu actin.tif]

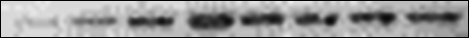

Supplement: S2 Fig — (ZIP) [file pone.0188932.s002.zip › support file fig.2/A/FaDu GRP78.tif]

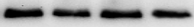

Supplement: S2 Fig — (ZIP) [file pone.0188932.s002.zip › support file fig.2/B/De actin.tif]

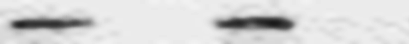

Supplement: S2 Fig — (ZIP) [file pone.0188932.s002.zip › support file fig.2/B/De GRP78.tif]

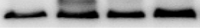

Supplement: S2 Fig — (ZIP) [file pone.0188932.s002.zip › support file fig.2/B/FaDu actin.tif]

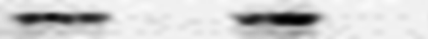

Supplement: S2 Fig — (ZIP) [file pone.0188932.s002.zip › support file fig.2/B/FaDu GRP78.tif]

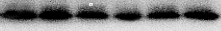

Supplement: S4 Fig — (ZIP) [file pone.0188932.s004.zip › support file fig4/B/De actin.tif]

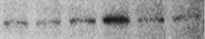

Supplement: S4 Fig — (ZIP) [file pone.0188932.s004.zip › support file fig4/B/De Bcl-2.tif]

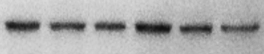

Supplement: S4 Fig — (ZIP) [file pone.0188932.s004.zip › support file fig4/B/De EGR1.tif]

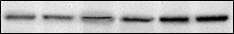

Supplement: S4 Fig — (ZIP) [file pone.0188932.s004.zip › support file fig4/B/De PARP.tif]

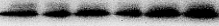

Supplement: S4 Fig — (ZIP) [file pone.0188932.s004.zip › support file fig4/B/FaDu actin.tif]

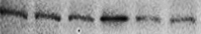

Supplement: S4 Fig — (ZIP) [file pone.0188932.s004.zip › support file fig4/B/FaDu Bcl-2.tif]

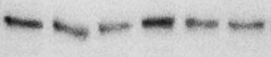

Supplement: S4 Fig — (ZIP) [file pone.0188932.s004.zip › support file fig4/B/FaDu EGR1.tif]

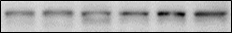

Supplement: S4 Fig — (ZIP) [file pone.0188932.s004.zip › support file fig4/B/FaDu PARP.tif]

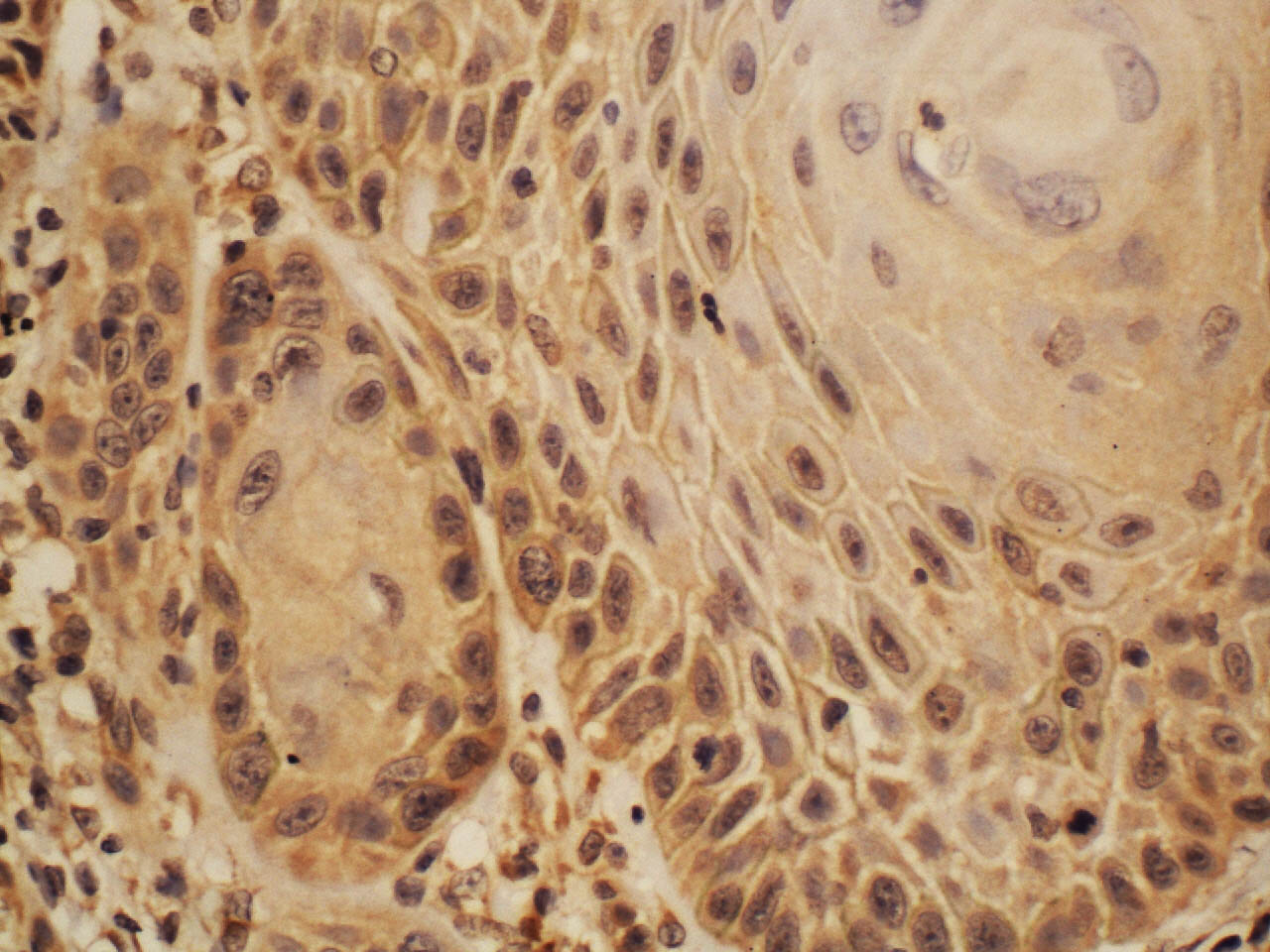

Supplement: S6 Fig — (ZIP) [file pone.0188932.s006.zip › support file fig.6/A/EGFR.jpg]

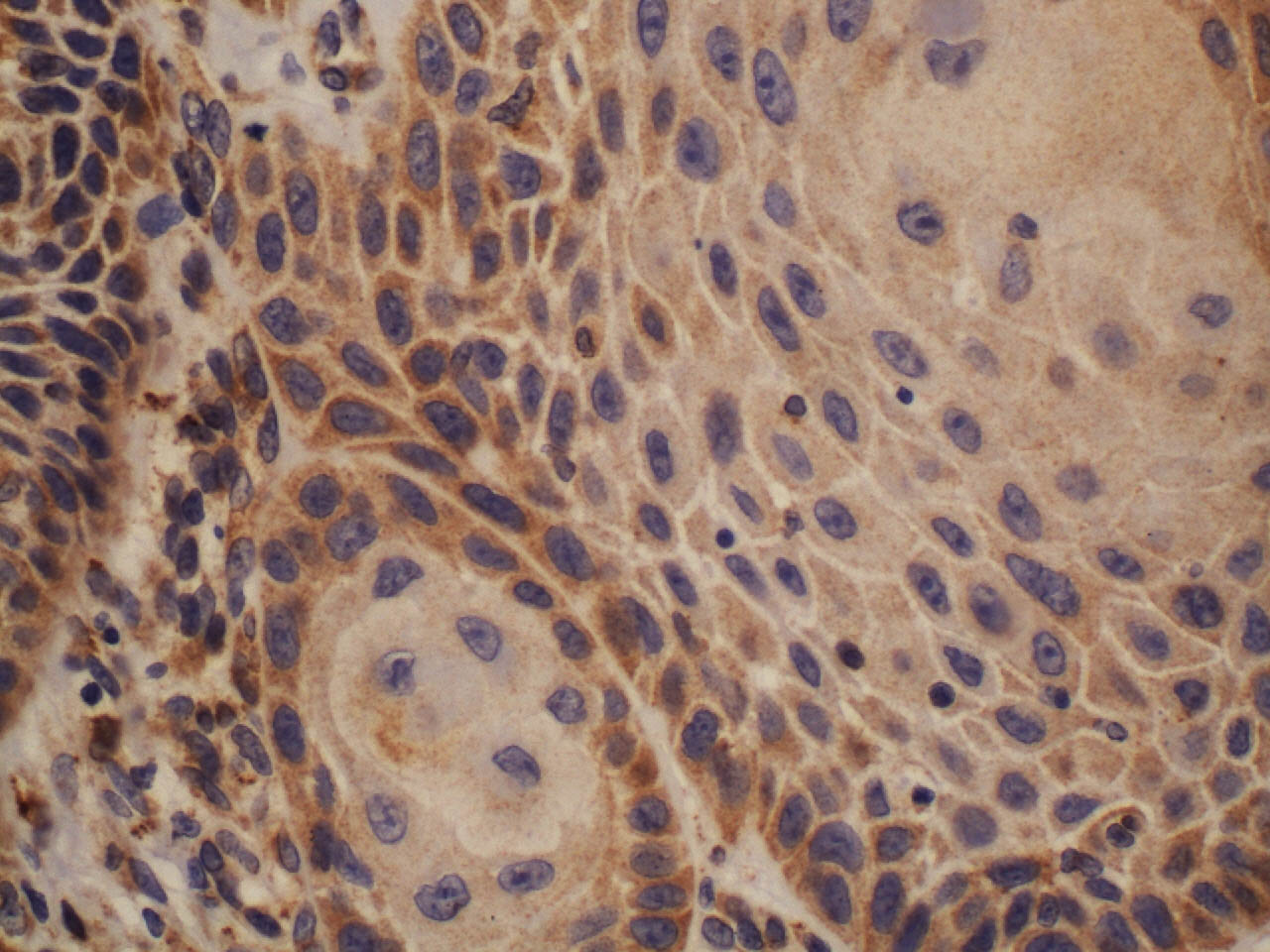

Supplement: S6 Fig — (ZIP) [file pone.0188932.s006.zip › support file fig.6/A/GRP78.jpg]

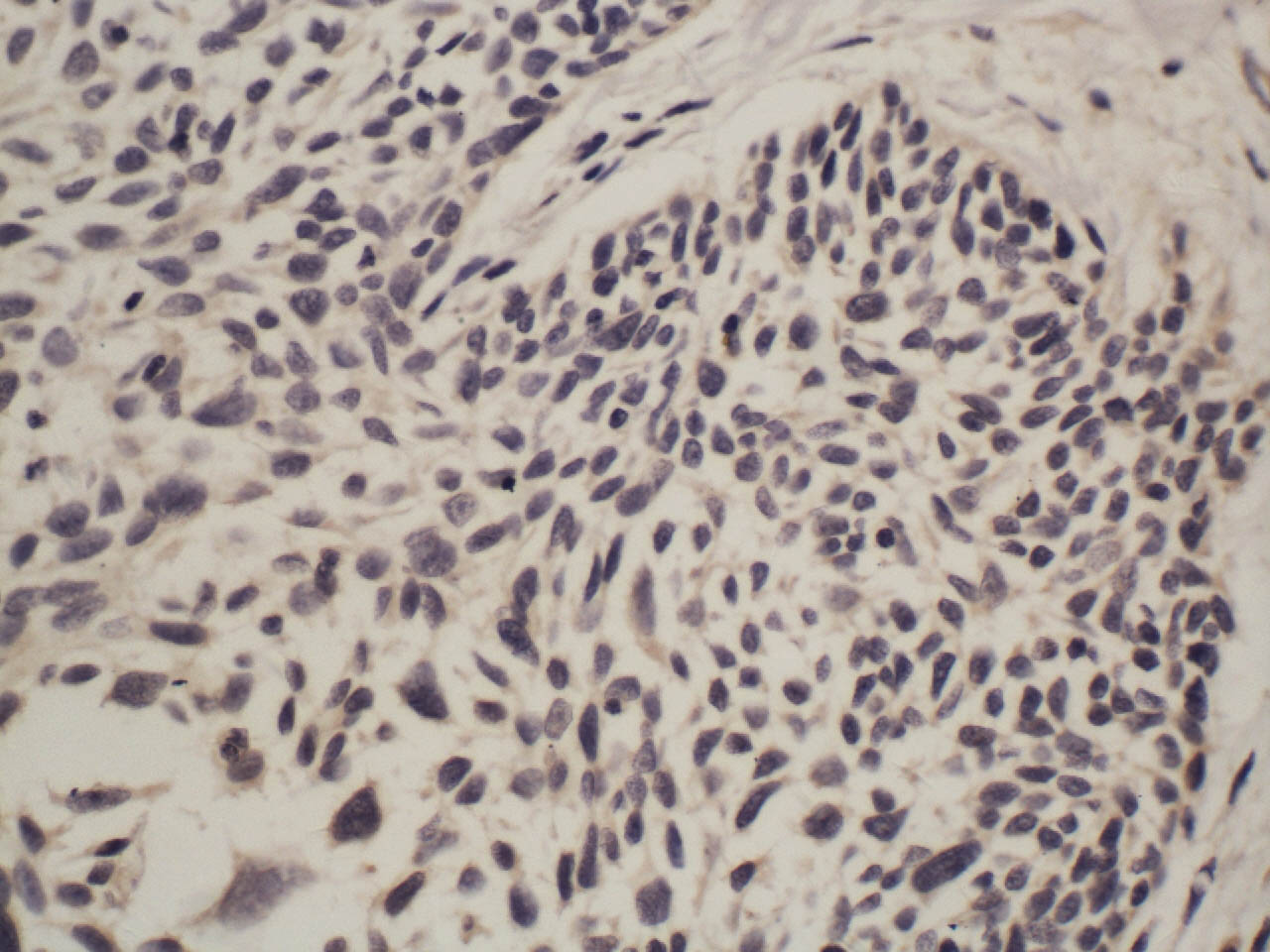

Supplement: S6 Fig — (ZIP) [file pone.0188932.s006.zip › support file fig.6/B/EGFR.jpg]

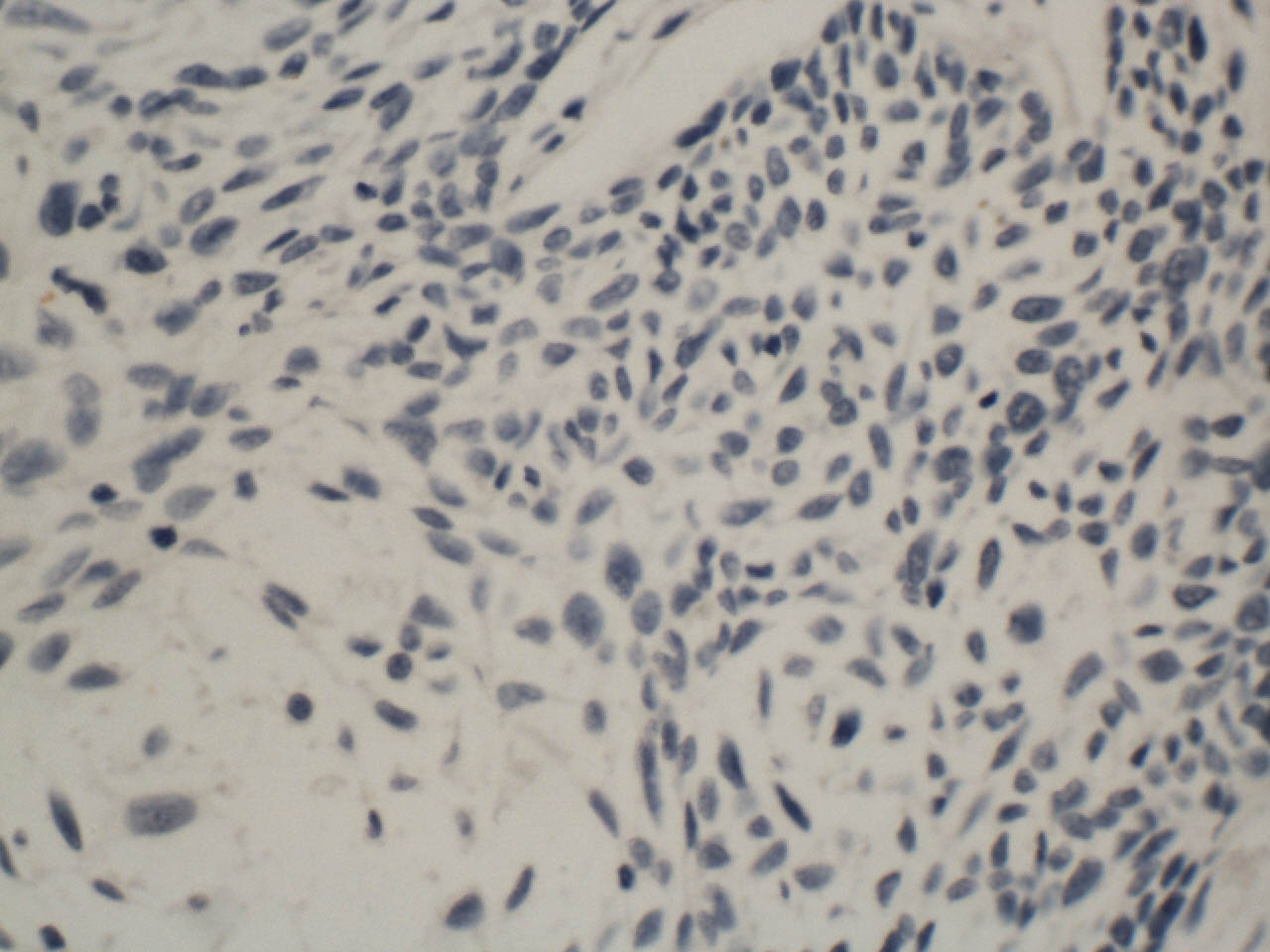

Supplement: S6 Fig — (ZIP) [file pone.0188932.s006.zip › support file fig.6/B/GRP78.jpg]
